# Cigarette Smoking and Mortality due to Stomach Cancer: Findings from the JACC Study.

**DOI:** 10.2188/jea.15.S113

**Published:** 2005-08-18

**Authors:** Yoshihisa Fujino, Tetsuya Mizoue, Noritaka Tokui, Shogo Kikuchi, Yoshihiro Hoshiyama, Hideaki Toyoshima, Hiroshi Yatsuya, Kiyomi Sakata, Akiko Tamakoshi, Reiko Ide, Tatsuhiko Kubo, Takesumi Yoshimura

**Affiliations:** 1 Department of Clinical Epidemiology, Institute of Industrial Ecological Sciences, University of Occupational and Environmental Health.; 2 Fukuoka Institute of Occupational Health.; 3 Department of Preventive Medicine, Graduate School of Medical, Sciences, Kyushu University.; 4 Department of Public Health, Aichi Medical University School of Medicine.; 5 Department of Public Health, Showa University School of Medicine.; 6 Department of Public Health/Health Information Dynamics, Nagoya University Graduate School of Medicine.; 7 Department of Public Health, Wakayama Medical University.; 8 Department of Preventive Medicine/Biostatistics and Medical Decision Making, Nagoya University Graduate School of Medicine.; 9 Fukuoka Institute of Health and Environmental Sciences.

**Keywords:** Stomach Neoplasms, Smoking, Cohort Studies, Japan, the JACC Study

## Abstract

BACKGROUND: Several epidemiologic studies reported the positive association between cigarette smoking and stomach cancer. The prevalence of smoking in men remains high in Japan compared to other developed countries. It is therefore of great importance to determine the impact of cigarette smoking on stomach cancer among the Japanese population. The Japan Collaborative Cohort Study (JACC Study) provided an opportunity to examine the association between smoking and the risk of mortality due to stomach cancer.

METHOD: A baseline survey was conducted throughout Japan from 1988 through 1990 among 110,792 inhabitants of 45 areas. Data retrieved for 98,062 participants (43,482 male and 54,580 female) who provided sufficient information about their smoking habits, without any history of caner at the baseline. Of total 970,251 person-years, 757deaths due to stomach caner were identified.

RESULTS: Current smokers were at a higher risk of death due to stomach cancer than non-smokers (Hazard ratio = 1.36; 95% confidence interval [CI]: 1.07, 1.73). The risk of stomach cancer for men who smoked 15 or more cigarettes per day was approximately 1.4-fold greater than that of non-smokers, and those who smoked 35 or more cigarettes per day had an approximately 1.7-fold higher risk of stomach cancer, although the dose-response trend among men was unclear (p for trend = 0.063). No associations between smoking and stomach cancer were detected among women.

CONCLUSION: The present results, together with previous findings, strongly support a hypothesis that cigarette smoking increases the risk of stomach cancer in Japanese men.

The incidence and mortality rates of stomach cancer are uniquely high among the Japanese population. Although it has been suggested that stomach cancer might be related to environmental factors,^1^ the reason for this trend in Japan remains unclear. According to the International Agency for Research on Cancer (IARC),^2^ numerous epidemiologic studies (both cohort and case control) conducted throughout the world have revealed a consistent association between stomach cancer and cigarette smoking. Furthermore, this correlation does not appear to be due to the confounding effects of alcohol consumption, *Helicobacter pylori* infection or dietary factors.

Previously, several cohort studies conducted in Japan consistently reported the positive association between cigarette smoking and stomach cancer.^3^^-^^10^ However, the knowledge obtained regarding the impact of cigarette smoking on stomach cancer is limited because most of these studies covered few areas of Japan.^3^^-^^5^^,^^7^^,^^9^^,^^10^ However, it is thought that stomach cancer is strongly related to environmental and ecological factors. The study that covered the widest areas is one reported by Hirayama,^8^ but the study was started in 1965 and followed-up for 17 years. During this period and until today, environmental conditions including dietary habits and cigarette smoking have dramatically changed in Japan. In addition, the prevalence of smoking is still high in Japan compared to other developed countries.^11^ Therefore, updated information is needed in regard to the nation wide impact of cigarette smoking on stomach cancer among the Japanese population. Furthermore, it is of particular interest to examine whether a small number of cigarettes increases the risk of stomach cancer because previous studies often used the cut-off point for 20 cigarettes per day.^3^^,^^5^^-^^7^^,^^10^

The Japan Collaborative Cohort Study (JACC Study) for Evaluation of Cancer Risk sponsored by the Ministry of Education, Science, Sports and Culture of Japan (Monbusho) involving 45 areas throughout Japan provided an opportunity to examine prospectively the association between smoking and the risk of mortality due to stomach cancer.

## METHODS

The details of the JACC study have been described elsewhere.^12^^-^^18^ Briefly, a baseline survey was conducted between 1988 and 1990. A total of 110,792 participants (46,465 men and 64,327 women) apparently healthy individuals who were aged between 40 and 79 years were enrolled into the study and completed the questionnaire. Twenty-four institutions from across the country participated in the study. The questionnaire addressed various health-related lifestyle choices, including smoking, alcohol consumption, occupation, diet and medical history. Follow-up surveys were conducted annually in order to determine the vital status of the participants. For deceased subjects, the cause of death was recorded from the official death certificate held at the relevant regional health centre, and was classified according to the International Classification of Disease, 10th Revision (ICD-10). Our analysis also included follow-up data that were collected before 1999. The informed consent procedures were approved by the Ethics Committee of Medical Care and Research at the University of Occupational and Environmental Health, Kitakyushu, Japan, and the Ethical Board of the Nagoya University School of Medicine, Japan. Deaths due to stomach cancer were defined according to category C16 of the ICD-10.

### Data Retrieval for Analysis

In order to identify the appropriate data for our analysis, we first restricted the dataset to include only those subjects who lacked a previous history of any type of cancer (n = 109,313). Our analysis was then further limited to the 98,062 participants (43,482 male and 54,580 female) who provided sufficient information about their smoking habits.

### Cigarette Smoking and Other Factors

The questionnaire required participants to indicate their smoking status as ‘non-smoker’, ‘past smoker’, or ‘current smoker’. For smokers, the number of cigarettes smoked per day was also recorded. The total number of ‘cigarette-years’ for smokers was calculated by multiplying the number of cigarettes smoked per day by the duration (years) of smoking. The following baseline characteristics, which might be related to stomach cancer, were also considered: age (5-year categories); alcohol consumption^9^^,^^19^ (‘current habitual drinker’, ‘previous habitual drinker’ or ‘teetotal’); consumption of green tea^20^ (‘almost every day’, ‘three times or more per week’, or ‘less than three times per week’); preference for salty foods^21^^-^^23^ (‘like salty foods’, ‘neutral towards salty foods’ or ‘dislike salty foods’); and educational level^15^^,^^24^^,^^25^ (‘attended school beyond the age of 18 years’, ‘attended school until the age of 16-17 years’ or ‘attended school until the age of 15 years or below’).

### Statistical Analysis

The Cox proportional-hazards regression model was used to estimate the hazard risk (HR) of smoking for mortality due to stomach cancer, while adjusting for the potential confounding factors listed above. Missing values in each variable were treated as an additional category in the analysis. All calculations were performed using the Statistical Analysis System^®^.^26^

## RESULTS

Table 1 shows the baseline characteristics of the study group according to smoking status. Subjects who did not smoke were less likely to consume alcohol. Current smokers were more likely to favour salty foods. Educational levels and green-tea consumption did not significantly differ between the three smoking groups: current smoker, past smoker or non-smoker. These trends were similar for both sexes.

**Table 1.** Baseline characteristics according to smoking status.

	Male			Female		
	
	Non-smoker	Past smoker	Current smoker	Non-smoker	Past smoker	Current smoker
	n = 9014	n = 11471	n = 22997	n = 9014	n = 11471	n = 22997
Age (year, mean and SD)	57.0 (10.3)	60.0 (10.1)	56.2 (10.0)	57.0 (10.3)	60.0 (10.1)	56.2 (10.0)

Percent of group						
Age (year) at leaving education (%)						
-15	27.1	26.2	27.8	27.1	26.2	27.8
16-17	13.7	14.2	14.5	13.7	14.2	14.5
18+	32.2	32.3	31.4	32.2	32.3	31.4
Missing	27.1	27.4	26.3	27.1	27.4	26.3

Alcohol consumption (%)						
Teetotal	24.8	16.1	16.8	24.8	16.1	16.8
Habitual	67.4	71.3	75.6	67.4	71.3	75.6
Past habitual	4.6	9.4	4.8	4.6	9.4	4.8
Missing	3.1	3.2	2.9	3.1	3.2	2.9

Preference for salty foods (%)						
Like	29.4	31.4	37.8	29.4	31.4	37.8
Normal	39.8	38.4	35.4	39.8	38.4	35.4
Dislike	13.3	10.8	8.5	13.3	10.8	8.5
Missing	17.5	19.4	18.3	17.5	19.4	18.3

Green-tea consumption (%)						
Every day	75.4	77.0	77.0	75.4	77.0	77.0
3+ times per week	4.7	3.9	4.5	4.7	3.9	4.5
less than 3 times per week	12.0	10.8	9.9	12.0	10.8	9.9
Missing	7.9	8.4	8.6	7.9	8.4	8.6

The risk of mortality due to stomach cancer according to smoking status in men is shown in Table 2. Current smokers were at a higher risk of death due to stomach cancer than were non-smokers (HR = 1.36; 95% confidence interval [CI]: 1.07, 1.73). The risk of mortality due to stomach cancer for men who smoked 15 or more cigarettes per day was approximately 1.4-fold greater than that of non-smokers. Although the dose-response trend among men was unclear (p for trend = 0.063), those who smoked 35 or more cigarettes per day had an approximately 1.7-fold higher risk of death due to stomach cancer. The HR of men who smoked between 1 and 14 cigarettes per day was not significantly increased (HR = 1.28; 95% CI: 0.93, 1.78). However, when this category was extended to include subjects who smoked 15 cigarettes per day, the HR was significantly greater (HR = 1.39; 95% CI: 1.04, 1.85). The multivariate models, which included alcohol consumption, preference for salty foods, green-tea consumption and educational levels, showed similar results to the age-adjusted models. No statistically significant associations between smoking and risk of mortality due to stomach cancer were detected among women in our study group (Table 3).

**Table 2.** Hazard ratios by smoking status in men.

	Person-years	No of aases	Age-adjusted	Multivariate*
	
			Hazard ratio (95% CI)	Hazard ratio (95% CI)
Non-smoker	89289	87	1.00 (reference)	1.00 (reference)
Past smoker	110972	164	1.25 (0.96 - 1.62)	1.24 (0.95 - 1.61)
Current smoker	223994	271	1.36 (1.07 - 1.73)	1.33 (1.04 - 1.70)

Number of cigarettes per day				
1 - 14	40826	62	1.28 (0.93 - 1.78)	1.27 (0.91 - 1.76)
15 - 24	122445	162	1.43 (1.10 - 1.86)	1.40 (1.08 - 1.83)
25 - 34	39162	27	1.03 (0.66 - 1.59)	1.01 (0.65 - 1.56)
35+	21561	20	1.70 (1.04 - 2.78)	1.64 (1.00 - 2.68)

* : Models included age, smoking, alcohol consumption, educational level, preference for salty foods and green-tea consumption.

CI : confidence interval.

**Table 3.** Hazard ratios by smoking status in women.

	Person-years	No of aases	Age-adjusted	Multivariate*
	
			Hazard ratio (95% CI)	Hazard ratio (95% CI)
Non-smoker	508462	220	1.00 (reference)	1.00 (reference)
Past smoker	8884	6	1.20 (0.53 - 2.70)	1.17 (0.51 - 2.65)
Current smoker	28650	9	0.77 (0.40 - 1.50)	0.77 (0.39 - 1.50)

Number of cigarettes per day				
1 - 14	15798	6	0.851 (0.38 - 1.92)	0.856 (0.38 - 1.94)
15 - 24	10738	3	0.757 (0.24 - 2.37)	0.738 (0.24 - 2.32)
25 - 34	1307	0	-	-
35+	808	0	-	-

* : Models included age, smoking, alcohol consumption, educational level, preference for salty foods and green-tea consumption.

CI : confidence interval.

Table 4 shows the HRs according to cigarette-years in men. Compared with non-smokers, men who had more than 400 cigarette-years had a 1.3- to 1.4-fold higher risk of death due to stomach cancer. No statistically significant associations between the number of cigarette-years and mortality due to stomach cancer were detected among women (Table 5).

**Table 4.** Hazard ratios according to cigarette-years in men.

	Person-years	No of aases	Age-adjusted	Multivariate*
	
			Hazard ratio (95% CI)	Hazard ratio (95% CI)
Non-smoker	89289	87	1.00 (reference)	1.00 (reference)
Past smoker	110972	164	1.22 (0.95 - 1.57)	1.23 (0.96 - 1.59)

Cigarette-years				
1 - 399	32443	30	1.36 (0.90 - 2.05)	1.38 (0.91 - 2.08)
400 - 799	107185	114	1.35 (1.03 - 1.77)	1.36 (1.04 - 1.79)
800+	74075	116	1.33 (1.01 - 1.74)	1.31 (1.00 - 1.72)

* : Models included age, smoking, alcohol consumption, educational level, preference for salty foods and green-tea consumption.

CI : confidence interval.

**Table 5.** Hazard ratios according to cigarette-years in women.

	Person-years	No of aases	Age-adjusted	Multivariate*
	
			Hazard ratio (95% CI)	Hazard ratio (95% CI)
Non-smoker	508462	220	1.00 (reference)	1.00 (reference)
Past smoker	8884	6	1.21 (0.54 - 2.72)	1.18 (0.52 - 2.67)

Cigarette-years				
1 - 399	18108	3	0.47 (0.15 - 1.45)	0.48 (0.15 - 1.49)
400 - 799	6918	6	1.85 (0.82 - 4.16)	1.79 (0.79 - 4.05)
800+	2007	0	-	-

* : Models included age, smoking, alcohol consumption, educational level, preference for salty foods and green-tea consumption.

CI : confidence interval.

## DISCUSSION

The present study showed that smoking increased the risk of mortality due to stomach cancer in men, although the dose-response relationship was unclear. Previous studies that have examined the dose-response relationship between cigarette smoking and stomach cancer have reported inconsistent results.^1^ The recent Japan Public Health Centre (JPHC) study was a detailed prospective analysis of the association between cigarette smoking and the occurrence of stomach cancer among the Japanese population.^9^ The JPHC study group reported that current smokers had a 1.7-fold higher risk of stomach cancer, although no obvious dose-response relationship was observed. In addition, the association was only significant for the differentiated type of stomach cancer. Koizumi reported a similar association from a pooled analysis of 2 population-based prospective cohort studies in Miyagi Prefecture^5^; the pooled RR of current smokers for stomach cancer occurrence was 1.77 (95% CI: 1.29, 2.43). The magnitude of the risk of smoking for stomach cancer in the present study was lower than those reported by these studies. However, this might be partly due to the fact that the present study used mortality data rather than incidence data. Another case-control study reported a significant dose-response relationship between smoking and stomach cancer according to the number of cigarette-years;^27^ this was not consistent with the present findings, although we did show that some categories of cigarette-year were associated with an increased risk of death due to stomach cancer in men.

Despite the lack of a clear dose-response relationship between cigarette smoking and stomach cancer, the fact that current smokers have an increased risk of mortality due to stomach cancer has important implications for public health, particularly as stomach cancer is relatively common in Japan.^11^ The incidence and mortality rate of stomach cancer differ substantially according to geographical area,^28^ which implies that stomach cancer might be strongly associated with environmental factors. However, the specific causes of this association remain unclear, with the exception of infection by Helicobacter pylori^29^ and possibly some dietary factors, such as consumption of salty foods.^21^^,^^23^ The JACC study failed to identify certain dietary factors that are strongly associated with stomach cancer among its subjects. These results therefore suggest that smoking might have a considerable influence on stomach cancer, at least among the Japanese population, in which the prevalence of smoking remains high (approximately 54% among males).^11^ Furthermore, the present study revealed that smoking even a relatively small number of cigarettes (1-15 cigarettes per day) significantly increased the risk for mortality due to stomach cancer. Therefore, in order to prevent stomach cancer, the overall negative effect of cigarette smoking should be strongly emphasized, regardless of the number of cigarettes that are smoked per day.

Several limitations of the present study should be mentioned. First, previous studies have suggested that the etiology of stomach cancer might differ between anatomical sites and histological types,^9^^,^^19^^,^^30^ however, we did not distinguish between these categories in the present study. Second, the smoking status and the number of cigarettes that an individual smokes per day might vary during different life stages; the present study analysed only baseline information, so the actual association might be even stronger than that identified using our dataset. Third, some lifestyle-related factors differ according to smoking status, and the present analysis might have omitted some such risk factors for stomach cancer. In fact, we found that current smokers were more likely to consume alcohol and to like salty foods, and were less likely to consume green tea, although adjusting these factors did not alter the results of our analysis. Perhaps more importantly, ecological level factors might be associated with the risk of stomach cancer, but these cannot be adequately assessed in investigations that are carried out at the level of the individual.^31^ Fourth, and finally, the distribution of the responses to the question ‘how many cigarettes do you consume per day’ should be treated with caution, because the participants tended to answer using boundary numbers (that is, 5, 10, 15, 20, 25, 30, 35, 40 or 45); this might have masked the true dose-response relationship among our data.

The present study reveals that cigarette smoking increases the risk of mortality due to stomach cancer in men. This relationship was robust even when an individual smoked less than 20 cigarettes per day. These results support a health policy stressing the harm that is caused to the human body through smoking. Furthermore, it is essential to raise public awareness in Japan that smoking increases not only the risk of lung cancer and other types of cancer but also, specifically, stomach cancer, as the incidence and mortality of stomach cancer remain high among the Japanese population.

## THE MEMBER LIST OF THE JACC STUDY GROUP

The present investigators involved, with the co-authorship of this paper, in the JACC Study and their affiliations are as follows: Dr. Akiko Tamakoshi (present chairman of the study group), Nagoya University Graduate School of Medicine; Dr. Mitsuru Mori, Sapporo Medical University School of Medicine; Dr. Yutaka Motohashi, Akita University School of Medicine; Dr. Ichiro Tsuji, Tohoku University Graduate School of Medicine; Dr. Yosikazu Nakamura, Jichi Medical School; Dr. Hiroyasu Iso, Institute of Community Medicine, University of Tsukuba; Dr. Haruo Mikami, Chiba Cancer Center; Dr. Yutaka Inaba, Juntendo University School of Medicine; Dr. Yoshiharu Hoshiyama, University of Human Arts and Sciences; Dr. Hiroshi Suzuki, Niigata University School of Medicine; Dr. Hiroyuki Shimizu, Gifu University School of Medicine; Dr. Hideaki Toyoshima, Nagoya University Graduate School of Medicine; Dr. Kenji Wakai, Aichi Cancer Center Research Institute; Dr. Shinkan Tokudome, Nagoya City University Graduate School of Medical Sciences; Dr. Yoshinori Ito, Fujita Health University School of Health Sciences; Dr. Shuji Hashimoto, Fujita Health University School of Medicine; Dr. Shogo Kikuchi, Aichi Medical University School of Medicine; Dr. Akio Koizumi, Graduate School of Medicine and Faculty of Medicine, Kyoto University; Dr. Takashi Kawamura, Kyoto University Center for Student Health; Dr. Yoshiyuki Watanabe, Kyoto Prefectural University of Medicine Graduate School of Medical Science; Dr. Tsuneharu Miki, Graduate School of Medical Science, Kyoto Prefectural University of Medicine; Dr. Chigusa Date, Faculty of Human Environmental Sciences, Mukogawa Women’s University ; Dr. Kiyomi Sakata, Wakayama Medical University; Dr. Takayuki Nose, Tottori University Faculty of Medicine; Dr. Norihiko Hayakawa, Research Institute for Radiation Biology and Medicine, Hiroshima University; Dr. Takesumi Yoshimura, Fukuoka Institute of Health and Environmental Sciences; Dr. Akira Shibata, Kurume University School of Medicine; Dr. Naoyuki Okamoto, Kanagawa Cancer Center; Dr. Hideo Shio, Moriyama Municipal Hospital; Dr. Yoshiyuki Ohno, Asahi Rosai Hospital; Dr. Tomoyuki Kitagawa, Cancer Institute of the Japanese Foundation for Cancer Research; Dr. Toshio Kuroki, Gifu University; and Dr. Kazuo Tajima, Aichi Cancer Center Research Institute.
